# Efficacy and Safety of Daratumumab, Pomalidomide, and Dexamethasone (DPd) Compared to Daratumumab, Bortezomib, and Dexamethasone (DVd) in Daratumumab–Naïve Relapsed Multiple Myeloma

**DOI:** 10.3390/cancers15194894

**Published:** 2023-10-09

**Authors:** Aimaz Afrough, Shebli Atrash, Barry Paul, Evguenia Ouchveridze, Nausheen Ahmed, Zahra Mahmoudjafari, Anam Bashir, Omar Alkharabsheh, Hamza Hashmi, Al-Ola Abdallah

**Affiliations:** 1 Hematologic Malignancies & Cellular Therapy Program, Simmons Comprehensive Cancer Center, UT Southwestern Medical Center, Dallas, TX 75390, USA; anam.bashir@utsouthwestern.edu; 2 US Myeloma Innovations Research Collaborative (USMIRC), Westwood, KS 66205, USA; shebli.atrash@atriumhealth.org (S.A.); barry.paul@atriumhealth.org (B.P.); nahmed5@kumc.edu (N.A.); zmahmoudjafari@kumc.edu (Z.M.); oalkharabsheh@health.southalabama.edu (O.A.); hashmih@musc.edu (H.H.); aabdallah@kumc.edu (A.-O.A.); 3 Levine Cancer Institute, Carolinas Healthcare System, Charlotte, NC 28204, USA; 4 Division of Hematologic Malignancies & Cellular Therapeutics, University of Kansas Medical Center, Westwood, KS 66160, USA; jenny.ouchveridze@gmail.com; 5 Division of Pharmacy, University of Kansas Medical Center, Westwood, KS 66160, USA; 6 Division of Hematology/Oncology, University of South Alabama Mitchell Cancer Institute, Mobile, AL 36604, USA; 7 Division of Hematology/Oncology, Medical University of South Carolina, Charleston, SC 29425, USA

**Keywords:** DPd, DVd, Daratumumab naïve, RRMM, outcome

## Abstract

**Simple Summary:**

We have been studying different combinations of medications to treat relapsed or refractory multiple myeloma. Among these combinations, one includes daratumumab with pomalidomide and dexamethasone (DPd), and another includes daratumumab with bortezomib and dexamethasone (DVd). So far, there have not been any direct comparisons performed through a clinical trial. In our analysis, we found that the DPd group had more patients with high-risk disease characteristics. Interestingly, the DPd group showed a better response to treatment. However, when we looked at the time until the disease progressed and overall survival, we found that these were similar between the two groups. It is important to note that we should be cautious in drawing conclusions from these findings because there were differences in the characteristics of the patients and lengths and durations of treatment, and the number of patients in both treatment groups was relatively small. Our study highlights the importance of considering factors like the type of patients, the side effects of the medications, and the characteristics of the disease when deciding which treatments to use. It is crucial to personalize the treatment approach for each individual based on these factors.

**Abstract:**

Daratumumab-based combinations with pomalidomide/dexamethasone (DPd), or bortezomib/dexamethasone (DVd), have shown activity in relapsed/refractory multiple myeloma (RRMM) patients. However, no direct comparisons of safety or efficacy of the two regimens have been published to date. We conducted a retrospective study to compare the safety and efficacy of DPd and DVd in daratumumab-naïve RRMM patients. We included 140 daratumumab-naïve patients who had received DPd or DVd for RRMM. Overall, the DPd group had a greater number of patients who had high-risk disease characteristics. Although response was deeper in the DPd group, the median progression-free survival (PFS) and overall survival (OS) were similar between the two groups. The DPd group exhibited a higher incidence of hematologic toxicities, whereas the DVd group had a higher incidence of peripheral neuropathy. The study results showed that while DPd may provide a deeper response, there was no significant difference in PFS or OS compared to DVd. For the high proportion of difficult-to-treat patients, duration of treatment may have contributed to these results, indicating that patient and disease characteristics should be considered when selecting salvage treatments.

## 1. Introduction

Daratumumab is a human immunoglobulin G1 kappa monoclonal antibody targeting CD38 originally approved for monotherapy treatment of relapsed refractory multiple myeloma (RRMM) in 2015. Subsequently, the addition of daratumumab to various backbones have yielded exceptional efficacy in RRMM, transplant-eligible, and transplant-ineligible newly diagnosed multiple myeloma (NDMM) patients [1,2,3,4]. However, triplet regimens such as VRd (bortezomib, lenalidomide, and dexamethasone), as well as VCd (bortezomib, cyclophosphamide, and dexamethasone) are still widely used induction regimens for NDMM in practice [5]. Therefore, a significant proportion of patients are not exposed to daratumumab in the frontline setting, and daratumumab remains an attractive option in subsequent lines of treatment.

Several daratumumab-based triplet regimens are commonly employed for relapsed myeloma, such as daratumumab, carfilzomib, and dexamethasone, (DKd); daratumumab, pomalidomide, and dexamethasone (DPd); daratumumab, bortezomib, and dexamethasone (DVd); or daratumumab lenalidomide and dexamethasone (DRd) [6]. The choice of combination depends on multiple factors, including prior therapies, exposure/refractoriness, patient comorbidities, route of administration, and patient preference. The increased utilization of lenalidomide in earlier lines of treatment, such as induction and maintenance, has led to an increased incidence of lenalidomide-refractory disease, which limits its utilization in subsequent lines of therapy [7,8]. In the treatment landscape of newly diagnosed and relapsed refractory multiple myeloma, there are a notable lack of robust real-world data comparing the effectiveness of different regimens. While some comparative data exist between DKd and DVd [9], a significant gap remains when it comes to comparing DPd, which incorporates a third-generation immunomodulatory agent (IMiD) with DVd. This void in data is particularly significant considering the distinctive toxicity profile and the intravenous (IV) administration route associated with carfilzomib in the DKd regimen. Importantly, not all patients are able or willing to undergo the DKd therapy due to these specific considerations. Notably, DVd obtained FDA approval for the treatment of RRMM with at least one prior line of treatment on 21 November 2016 [10] based on the results of the phase 3 CASTOR trial [2]. On 16 June 2017, IV daratumumab in combination with pomalidomide and dexamethasone (Pd) received approval for RRMM patients who underwent at least two prior therapies, including lenalidomide and a proteasome inhibitor (PI) [11], based on the results of the phase 1b EQUULEUS trials [12]. Following this, the phase 3 APOLLO trial compared both IV and subcutaneous form of daratumumab in combination with Pd versus Pd alone in patients with RRMM and one prior line of treatment (LOT), including lenalidomide and a PI [4]. The favorable results of this trial led to the expansion of approval for use in an earlier line of treatment, achieved on 3 August 2021 [13]. Without head-to-head clinical trials comparing triplet regimens in this setting, retrospective studies can provide valuable insights into the real-world effectiveness of different treatment regimens. Many patients studied in these retrospective analyses would have been ineligible to receive these chemotherapy combinations in the clinical trials due to comorbidities, frailty, and organ dysfunction and hence are an accurate reflection of the real-world patient population. Moreover, these real-world analyses also provide valuable information about the utilization patterns, chemotherapy dosing and schedule, and toxicity management of these regimens in everyday clinical practice. Therefore, we conducted this single-center, retrospective analysis to evaluate the outcomes of DPd versus DVd in the relapsed MM patients. 

## 2. Materials and Methods

### 2.1. Study Population

This single center retrospective study was conducted at the Kansas University Medical Center (KUMC) in collaboration with the United States Myeloma Innovations Research Collaborative (USMIRC) after receiving approval from the KUMC Institutional Review Board. For this retrospective analysis, we identified 140 consecutive RRMM patients who had received either DPd or DVd for RRMM at the University of Kansas Cancer Center between January 2015 and June 2022. High-risk MM was defined based on the 2016 International Myeloma Working Group Criteria (IMWG), with the presence of t (4;14), del(17/17p), t (14;16), t (14;20), and gain(1q) by fluorescence in situ hybridization (FISH) at the time of diagnosis [14]. 

### 2.2. Treatment

The determination of treatment type was influenced by a range of factors. These factors encompassed various elements such as the timeline of FDA approvals for the specific line of treatment, the patient’s performance status, renal function, comorbidities, and the treating physician’s discretion. 

DPd was given as daratumumab at 16 mg/kg IV or 1800 mg SQ weekly for cycles 1 and 2, every 2 weeks for cycles 3–6, and then every 4 weeks afterwards; pomalidomide was dosed at 4 mg orally on days 1–21 of a 28-day cycle; and dexamethasone was given at 20 or 40 mg weekly. The DVd group received daratumumab 16 mg/kg IV or 1800 mg SQ weekly for 3 cycles, every 3 weeks for cycles 4–8, and then every 4 weeks afterwards, bortezomib (1.3 mg/m^2^) on days 1, 4, 8, and 11 every 3 weeks for 8 cycles, and dexamethasone 20 or 40 mg weekly in combination with daratumumab. Responses were evaluated using IMWG criteria [15], and toxicities were determined using common terminology criteria for adverse events v3.0 (CTCAE) grading [16]. Chemotherapy dosing and frequency were adjusted based on toxicities using package insert. The hematologic and non-hematologic toxicity profiles of both regimens were evaluated and compared. 

### 2.3. Outcome

We evaluated the overall disease response, progression-free survival (PFS), overall survival (OS), and duration of response (DOR). PFS was defined as the time from the initiation of treatment (DPd or DVd) to progression or death (regardless of the cause of death), whichever occurred first. OS was defined as the time from initiation of treatment (DPd or DVd) until death or last follow-up. DOR was defined as the time from first observation of partial response (PR) to the time of disease progression, with deaths from causes other than progression censored [17]. 

### 2.4. Statistical Analysis

For the overall cohort, comparisons between subgroups of interest were performed using Fisher’s exact tests for categorical variables and unequal variance *t*-tests for continuous variables. Unadjusted survival distributions were estimated using the Kaplan–Meier method, and comparisons were performed with the log-rank test. Univariable Cox proportional hazards regression models were used to evaluate the associations between survival outcomes and covariates; multivariable Cox proportional hazards regression models were fitted with pre-specified covariates of the Revised International Staging System (R-ISS) stage, cytogenetic risk, presence of extramedullary disease (EMD), history of autologous stem cell transplantation (ASCT), number of prior regimens, current treatment (DPd vs. DVd), double refractory status (both PI and IMiD), and response to treatment (very good partial response/partial response (VGPR/PR) vs. stringent complete response/complete response (sCR/CR)). A nearest matching analysis was used to compare two treatment groups, specifically matching for high-risk cytogenetics and EMD. We used the software R (Vienna, Austria) v2.15.1 and the survival package for the Kaplan–Meier method. For other statistical calculations, we employed the statistical software IBM SPSS (version 29.0). 

## 3. Results

### 3.1. Patients and Disease Characteristics

The study population consisted of 140 patients, with 97 treated with DPd, and 43 treated with DVd. Patient and disease characteristics are summarized in Table 1. The overall median follow-up was 22.5 months (range 1–78), with a median follow up of 24 (range 1–78) and 17 (range 1–49) months in the DPd and DVd groups, respectively (*p* = 0.143). 

The DPd group had more patients with high-risk disease features, such as high-risk cytogenetic abnormalities, EMD, prior ASCT, a higher number of prior regimens, and higher rate of double refractory disease, compared to the DVd group.

EMD was present in 28% of DPd compared to 9% in the DVd group (*p* = 0.015). Cytogenetic studies by FISH were available for 98% (137/140) of the patients, 60% (83/140) of whom had high-risk cytogenetic abnormalities, with 67% in the DPd and 45% in the DVd groups, respectively (*p* = 0.022). The median previous LOT was 2 (1–6), and 1 (1–5) in the DPd and DVd groups, respectively (*p* = 0.02). Of those who received DPd, 61% had double refractory disease, compared to 30% in the DVd group (*p* < 0.001). The proportion of lenalidomide-refractory patients was 82% in the DPd group compared to 51% in the DVd group (*p* < 0.001). There was no difference in the bortezomib refractoriness between the two treatment groups, with 53% in the DPd compared to 35% in the DVd group (*p* = 0.067). The incidence of pomalidomide refractoriness was 0% in DPd compared to 9% in the DVd group (*p* = 0.008). The incidence of carfilzomib refractoriness was 26% in DPd compared to 9% in the DVd group (*p* = 0.040).

### 3.2. Efficacy and Safety Outcome

The median duration of therapy was 10 (1–64) months in DPd, compared to 6 (1–42) months in the DVd group (*p* = 0.135).

The overall response rate (ORR) was 74% (103/140) for the entire patient population (74% in DPd group and 72% in DVd group). The median time to best response was 2 (1–9) months in the DPd, compared to 2 (1–6) in the DVd group (*p* = 0.736). VGPR or better response was seen in 47% and 28% in the DPd and the DVd groups, respectively (*p* = 0.041). Similarly, sCR and CR were seen in 37% and 14% in the DPd and the DVd groups, respectively (*p* = 0.005) (Table 2). The median DOR was 20.7 (95% CI, 14.9–37.1) and 15.6 (95% CI, 8.4–NA) months for the DPd and the DVd groups, respectively (hazard ratio (HR) = 1.2; 95% CI, 0.7–2.06; *p* = 0.5).

The most common adverse events related to the treatment (DPd vs. DVd) are summarized in Table 3. The most commonly reported grade 3 or higher adverse events for DPd and DVd were thrombocytopenia (12% vs. 40%), anemia (21% vs. 9%), neutropenia (74% vs. 14%), neutropenic fever (9% vs. 0%), and peripheral neuropathy (2% vs. 23%). The most common cause of treatment discontinuation was disease progression, followed by other adverse events, including 9% of patients receiving DVd discontinuing treatment due to peripheral neuropathy, as highlighted in Table 2. 

### 3.3. Survival Outcomes

**Figure 1:**
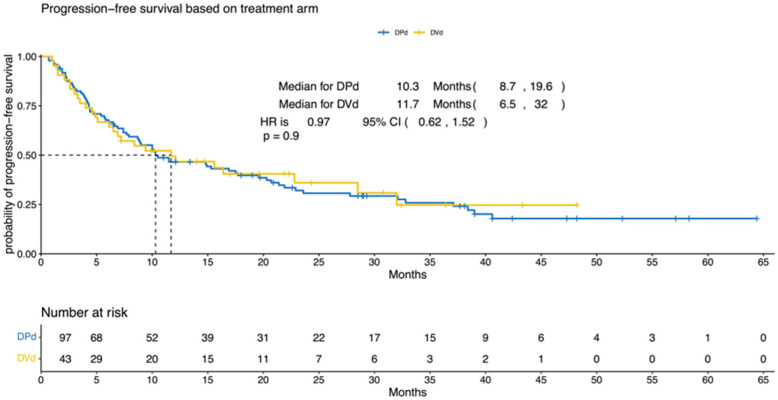
Progression-free survival according to treatment (DPd vs. DVd). Kaplan–Meier curves for the progression-free survival. PFS = progression-free survival, CI = confidence interval, HR = hazard ratio, DPd = daratumumab, pomalidomide, and dexamethasone, DVd = daratumumab, bortezomib, and dexamethasone.

**Figure 2:**
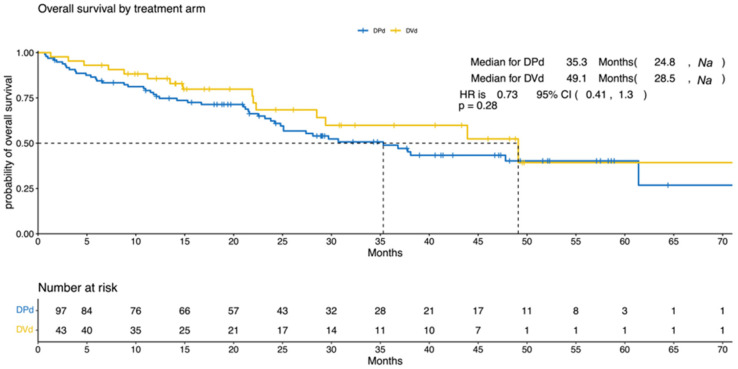
Overall survival according to treatment (DPd vs. DVd). Kaplan–Meier curves for overall survival. OS = overall survival, CI = confidence interval, HR = hazard ratio, DPd = daratumumab, pomalidomide, and dexamethasone, DVd = daratumumab, bortezomib, and dexamethasone.

The median PFS was 10.3 (95% CI, 8.7–19.6) and 11.7 (95% CI, 6.5–32) months for the DPd and the DVd groups, respectively (*p* = 0.9; HR = 0.97; 95% CI, 0.62–1.52; Figure 1). The median OS was 35.3 (95% CI, 24.8–NA) and 49.1 (95% CI, 28.5–NA) months for the DPd and the DVd groups, respectively (*p* = 0.284; HR = 0.73; 95% CI, 0.41–1.3; Figure 2). 

We performed a subgroup analysis to examine the outcomes of DPd and DVd treatments specifically in lenalidomide-refractory patients. In this subgroup, the median PFS was found to be 10.1 months for the DPd group, and 6.4 months for the DVd group (*p* = 0.58), indicating no significant difference between the two treatments in terms of PFS in lenalidomide-refractory patients. Similarly, when we analyzed the subgroup of bortezomib-refractory patients, we observed no significant difference in median PFS between the DPd and DVd treatments. The median PFS was 8.8 months for the DPd group and 6.8 months for the DVd group.

#### 3.3.1. Nearest Matching Analysis 

**Figure 3:**
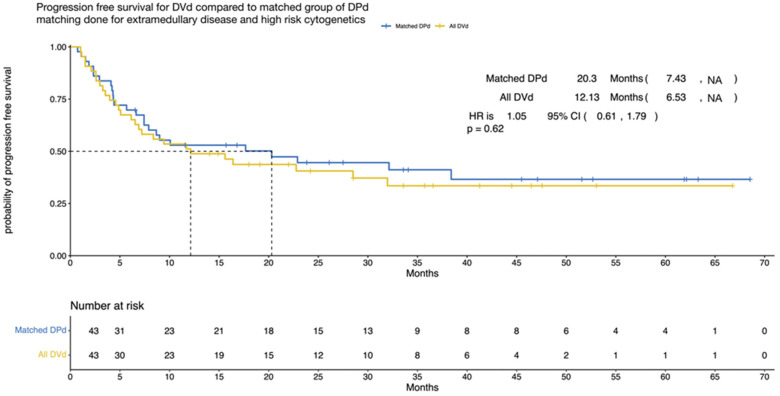
Progression-free survival (nearest matching) according to treatment (DPd vs. DVd). Kaplan–Meier curves for the progression-free survival. PFS = progression-free survival, CI = confidence interval, HR = hazard ratio, DPd = daratumumab, pomalidomide, and dexamethasone, DVd = daratumumab, bortezomib, and dexamethasone.

Through a 1:1 matching analysis, we decreased the sample size to n = 86 (43 in each group), revealing a median PFS of 20 months for the DPd group and 12 months for the DVd group. However, when comparing the two groups, we obtained a non-significant *p*-value of 0.62 (HR = 1.05; 95% CI, 0.61–1.79), as shown in Figure 3. This lack of statistical significance was likely due to the limited sample size in our study.

#### 3.3.2. Factors Predicting Survival

Cox regression analysis was conducted to identify the predictors of PFS and OS. The variables included in the analysis were R-ISS stage, cytogenetic risk, EMD, history of ASCT, number of prior regimens, current treatment (DPd vs. DVd), double refractory status, and response to treatment (VGPR/PR vs. sCR/CR). 

In the multivariate analysis for PFS in the entire cohort, R-ISS II (*p* = 0.046; HR = 1.80; 95% CI, 1.01–3.22), R-ISS III (*p* =< 0.001; HR = 3.13; 95% CI, 1.78–5.53), the presence of EMD (*p* = 0.013; HR = 1.94; 95% CI, 1.15–3.26), higher number of prior regimens (*p* = 0.01; HR = 1.33; 95% CI, 1.06–1.66), and response of VGPR/PR (*p* =< 0.001; HR = 4.47; 95% CI, 2.54–7.88) emerged as significant predictors of inferior PFS. On multivariate analysis for OS in the entire cohort, R-ISS III (*p* = 0.005; HR = 2.87; 95% CI, 1.37–5.99), greater number of prior regimens (*p* = 0.009; HR = 1.42; 95% CI, 1.09–1.85), and response of VGPR/PR (*p* = 0.003; HR = 3.01; 95% CI, 1.46–6.24) emerged as significant predictors of inferior OS (Table 4). The type of treatment (DPd vs. DVd) was not statistically significant in predicting PFS or OS.

## 4. Discussion

Multiple myeloma is a complex and challenging hematologic malignancy that requires a multifaceted treatment approach. Current therapeutic strategies in newly diagnosed multiple myeloma often include triplet therapy with a PI, IMiD, and steroid, followed by ASCT and maintenance therapy. This strategy improved 5-year survival to about 81% in the lenalidomide maintenance group compared to 61.5% in those who did not receive maintenance therapy [18]. Despite these advances, relapse is inevitable [19]. The efficacy and favorable toxicity profile of daratumumab has led to its widespread use in earlier lines of treatment, including newly diagnosed and early relapse settings. The choice of treatment regimen in RRMM depends on several factors, including previous treatments, comorbidities, and patient preference [20,21]. In the absence of head-to-head clinical trials comparing triplet regimens in this setting, we conducted a retrospective study to compare the “real world” results of DPd regimen, with DVd. 

In this study, treatment with DPd was associated with a deeper response; however, this did not translate into any differences in PFS or OS between the two treatment groups, after a median follow-up of 22.5 months. Several factors could have contributed to the lack of survival benefit with DPd, including the presence of more difficult-to-treat patients in the DPd group, such as those with high-risk cytogenetics, EMD, and double refractory disease, as well as those who had received a higher number of previous lines of treatment. As anticipated, the DPd group exhibited a higher incidence of neutropenia, whereas the DVd group had a greater incidence of peripheral neuropathy. However, the main reason for discontinuing treatment in both groups was disease progression. However, given that one-fourth of the patients developed grade 3 or higher peripheral neuropathy, and nearly 10% discontinued therapy due to this toxicity, patients with baseline peripheral neuropathy should avoid DVd for relapsed disease. Our findings are consistent with the results of previous clinical trials evaluating the efficacy of DPd or DVd in comparison to their doublet counterparts [2,4]. The EQUULEUS phase 1b study evaluated the safety and tolerability of daratumumab in combination with various treatment regimens. The report in outcome of 103 patient with RRMM with ≥2 prior lines of therapy who received daratumumab IV plus Pd showed the ORR of 60%. With median follow up of 13.1 months, the median PFS was 8.8 months, and median OS was 17.5 months [12]. The phase 3 APOLLO study investigated the outcomes of 304 RRMM patients who received Pd with or without daratumumab. Of the enrolled subjects, 79% in the DPd group and 80% in the Pd group were lenalidomide refractory, and about one-third of patients had high risk cytogenetic abnormalities (38% in DPd vs. 32% in Pd group). At a median follow-up of 16.9 months, the DPd showed longer PFS compared to Pd in the intention-to-treat (ITT) population (median PFS = 12.4 vs. 6.9 months, HR = 0.63, 95% CI = 0.47–0.85), as well as lenalidomide-refractory population [4]. Similarly, our retrospective study showed median PFS of 10.16 months (95% CI, 4.74–15.58). In the recent update from the APOLLO study, which had a median follow-up of 39.6 months [22], the median OS was reported as 34.4 months (95% CI, 23.7–40.3). Our study, on the other hand, showed a comparable median OS of 35.3 months (95% CI, 24.8–NA). These findings indicate that DPd treatment may offer a similar level of survival benefit as seen in previous clinical trials, even when considering the variations in patient populations and study designs.

The phase 3 CASTOR study investigated the outcomes of 498 RRMM patients who received bortezomib and dexamethasone (Vd) with or without daratumumab. Of the enrolled subjects, 24% in the DVd group and 33% in the Vd group were lenalidomide refractory, and about a fifth of patients had high risk cytogenetic abnormalities (22% in DVd vs. 21% in Vd group) [2]. At a median follow-up of 40.0 months, the DVd showed longer PFS compared to Vd in the ITT population (median PFS = 16.7 vs. 7.1 months, HR = 0.31, 95% CI = 0.25–0.40). However, the median PFS was significantly lower in lenalidomide-refractory population (median PFS = 7.8 vs. 4.9 months, HR = 0.44, 95% CI = 0.28–0.68) in comparison to the ITT population [23], suggesting that DVd may not be optimal for the lenalidomide-refractory population. 

In our study, 73% of the population was lenalidomide refractory, with a higher percentage of lenalidomide-refractory patients observed in DPd group (82%) compared to DVd group (51%). We found that the PFS, in the lenalidomide-refractory population was numerically superior in the DPd group (median PFS = 10.1 vs. 6.4 months; *p* = 0.58), but the difference was not statistically significant. Based on previously published data [24], patients with duration of prior lenalidomide treatment of less than two years, last lenalidomide dose of greater than 10 mg, R-ISS stage II/III, and high-risk cytogenetics seem to derive less benefit from DPd. Several of these findings were confirmed with our study, including advanced stage disease and high-risk cytogenetics being associated with worse PFS and OS. DKd for patients with RRMM was studied in the CANDOR trial [25], where DKd was associated with a median PFS of 28 months with treatment related mortality (TRM) of about 10%. With a reasonable PFS benefit in all categories, whether DKd is a preferred option for patients with these high-risk features remains an important clinical question. 

Like our result, the indirect comparison of DPd from APOLLO study with DVd from CASTOR study [26] showed no difference in terms of PFS between these treatment groups (HR 1.26; 95% CI, 0.99–1.60 in DPd, and HR 0.83; 95% CI, 0.63–1.10 in DVd group; *p*  =  0.20). However, after conducting cardinality matching, the DPd group showed a significant improvement in PFS with a 45% reduction in the risk of progression compared to the DVd group (HR = 0.55; 95% CI, 0.36–0.82; *p* < 0.01). It is important to note that indirect comparisons have limitations and should be interpreted cautiously. In our study, we employed nearest matching analysis, specifically matching for high-risk cytogenetics and EMD, revealing a median PFS of 20 months for DPd compared to 12 months in DVd. However, the nonsignificant *p*-value (*p* = 0.62) may be attributed to the limited sample size. Our study has several limitations that are inherent to a single-center retrospective analysis, including selection bias and missing data. However, this is the first study in literature reporting outcomes of the real-world use of DPd vs. DVd for patients with RRMM. Another limitation is the imbalanced distribution of patients between the two treatment groups, with a higher proportion of difficult-to-treat individuals in the DPd group. While the choice of therapy at the time of relapse is affected by several factors, including patient comorbidities, performance status, previous anti-myeloma therapies, as well as efficacy and toxicity profile of the next therapy under consideration, the precise reasons for choice of regimen in our patient population remain unknown due to the retrospective nature of the study. This imbalance in patient characteristics has the potential to bias the efficacy outcomes of the chosen regimen. Therefore, caution should be exercised when extrapolating the findings of our study to other populations or treatment settings. Given the study’s retrospective design, no data are available on the quality-of-life outcomes to determine the impact of DPd and DVd on symptom burden in patients receiving treatment. Multicenter and registry-based retrospective analyses with larger patient populations are needed to confirm the findings of our report and allow for more meaningful and statistically significant subgroup analyses. Similarly, well-designed randomized controlled trials with longer follow-ups are needed to clearly establish the superiority of one salvage regimen over the other. 

## 5. Conclusions

In conclusion, this single-center retrospective analysis found that DPd salvage treatment yielded deeper responses than the DVd regimen, without significant differences in PFS or OS. Both therapies were overall well tolerated. While DPd was associated with more hematological toxicities, DVd showed a higher incidence of peripheral neuropathy. In the treatment landscape of RRMM, the abundance of available therapies underscores the necessity of careful selection and sequencing of various salvage chemotherapy regimens. This study highlights the importance of meticulously considering patient demographics, toxicity profiles, and disease characteristics in selecting salvage treatments for RRMM. Furthermore, assessing patient-reported outcomes and quality of life measures could provide a holistic view of the treatment experience. Additional investigations into potential predictive biomarkers for treatment response and toxicity hold the promise of greatly enhancing treatment selection and customization strategies.

## Figures and Tables

**Table 1:** Patient Characteristics According to Therapy (DPd versus DVd).

Characteristic	N	Overall	DPd	DVd	*p*-Value
		(n = 140)	(n = 97; 69%)	(n = 43; 31%)	
Median age at diagnosis (range), years	140	66 (38–84)	66 (42–84)	68 (38–83)	0.338
<65		56 (40)	41 (42)	15 (35)	0.458
≥65		84 (60)	56 (58)	28 (65)	
Median follow-up (range), months	140	22 (1–78)	22 (1–78)	17 (1–49)	0.464
Gender	140				0.357
Female		62 (44)	40 (41)	22 (51)	
Male		78 (56)	57 (59)	21 (49)	
Race	140				0.854
White		108 (77)	74 (76)	34 (79)	
Black or African American		27 (19)	19 (20)	8 (19)	
Other		5 (4)	4 (4)	1 (2)	
Myeloma type, n (%)	140				0.774
IgA		35 (25)	26 (27)	9 (21)	
IgG		87 (62)	60 (62)	27 (63)	
IgM		2 (1)	1 (1)	1 (2)	
Light chain only		16 (12)	10 (10)	6 (14)	
Light-chain type, n (%)	140				1.0
Kappa		98 (70)	68 (70)	30 (70)	
Lambda		42 (30)	29 (30)	13 (30)	
R-ISS stage, n (%)	133				0.812
I		41 (31)	30 (32)	11 (27)	
II		50 (37.5)	35 (38)	15 (36.5)	
III		42 (31.5)	28 (30)	15 (36.5)	
Cytogenetics (FISH), n (%)	137				0.022
Standard risk		54 (39)	31 (33)	23 (55)	
High-risk		83 (60)	64 (67)	19 (45)	
High-risk cytogenetic	137				0.02
0		54 (39)	31 (32.5)	23 (55)	
1		41 (30)	29 (30.5)	12 (28.5)	
2		42 (31)	35 (37)	7 (16.5)	
Extramedullary disease	140				0.015
yes		31 (22)	27 (28)	4 (9)	
no		109 (78)	70 (72)	39 (91)	
Previous Autologous stem cell transplant	140				<0.001
yes		100 (71)	78 (80)	22 (51)	
no		40 (29)	19 (20)	21 (49)	
Number of Autologous stem cell transplant	140	1 (0–3)	1 (0–3)	1 (0–2)	0.317
Prior line of treatment, median (range)	140	2 (1–6)	2 (1–6)	1 (1–5)	0.002
Bortezomib Exposure, n (%)	140	133 (95)	93 (96)	40 (93)	0.675
Bortezomib Refractory, n (%)	140	66 (47)	51 (53)	15 (35)	0.067
Carfilzomib Exposure, n (%)	140	40 (29)	34 (35)	6 (14)	0.014
Carfilzomib Refractory, n (%)	140	29 (21)	25 (26)	4 (9)	0.04
Lenalidomide Exposure, n (%)	140	130 (93)	94 (97)	36 (84)	0.01
Lenalidomide Refractory, n (%)	140	102 (73)	80 (82)	22 (51)	<0.001
Pomalidomide Exposure, n (%)	140	16 (11)	12 (12)	4 (9)	0.776
Pomalidomide Refractory, n (%)	140	4 (3)	0 (0)	4 (9)	0.008
Double Refractory, n (%)	140	72 (51)	59 (61)	13 (30)	<0.001
Duration of treatment, median (rang), month	140	8 (1–64)	10 (1–64)	6 (1–42)	0.067

**Table 2:** Outcome According to Therapy (DPd versus DVd).

Characteristic	Overall	DPd	DVd	*p*-Value
Best response post treatment > VGPR	42 (30%)	36 (37%)	6 (14%)	0.005
Best response post treatment				
sCR + CR	42 (30%)	36 (37%)	6 (14%)	
VGPR	16 (11%)	10 (10%)	6 (14%)	
PR	45 (32%)	26 (27%)	19 (44%)	
SD + PD	37 (26%)	25 (26%)	12 (28%)	
Cumulative incidence of relapse 2-year (95% CI)	68.5% (60–77)	70% (60–79)	66% (58–74)	
Cause of treatment discontinuation				
Disease progression	99 (91%)	70 (95%)	29 (83%)	
Hematologic toxicity	2 (2%)	2 (3%)	0 (0%)	
Neuropathy	4 (4%)	1 (1%)	3 (9%)	
Infection	3 (3%)	1 (1%)	2 (6%)	
Cardiac event	1 (1%)	0 (0%)	1 (3%)	
PFS (mo), median (95% CI)	10.5 (8.7–17.7)	10.3 (8.7–19.6)	11.7 (6.5–32)	0.9
OS (mo), Median (95% CI)	37.8 (28.1–NA)	35.3 (24.8–NA)	49.1 (28.5–NA)	0.28

Not all percentages add up to 100% because of rounding. CR indicated complete remission; sCR, stringent CR; VGPR, very good partial response; PR, partial response; SD, stable disease; PD, progressive disease; PFS, progression-free survival; OS, overall survival; CI, confidence interval; mo, months.

**Table 3:** Most Common Treatment Related Adverse Events v3.0 (grade 3/4) According to Therapy (DPd vs. DVd).

	Overall	DPd	DVd
Treatment-related grade ≥ 3 hematologic toxicity			
Leukopenia	62 (44%)	57 (59%)	5 (12%)
Neutropenia	78 (56%)	72 (74%)	6 (14%)
Lymphopenia	66 (47%)	51 (53%)	15 (35%)
Anemia	24 (17%)	20 (21%)	4 (9%)
Thrombocytopenia	29 (21%)	12 (12%)	17 (40%)
Treatment-related grade ≥ 3 non-hematologic toxicity			
GI symptoms (Nausea, vomiting, diarrhea)	0 (0%)	0 (0%)	0 (0%)
Elevated liver enzymes	0 (0%)	0 (0%)	0 (0%)
Peripheral neuropathy	12 (9%)	2 (2%)	10 (23%)
Venous thromboembolism	0 (0%)	0 (0%)	0 (0%)
Neutropenic fever	4 (3%)	4 (9%)	0 (0%)
Infusion related reaction	0 (0%)	0 (0%)	0 (0%)

**Table 4:** Multivariate analysis of PFS and OS.

Variables	PFS		OS	
	Multivariate		Multivariate	
	HR (95%CI)	*p*-Value	HR (95%CI)	*p*-Value
R-ISS		<0.001		0.009
I	Ref.		Ref.	
II	1.80 (1.01–3.22)	0.04	1.36 (0.63–2.92)	0.43
III	3.13 (1.78–5.53)	<0.001	2.87 (1.3–5.99)	0.005
Cytogenetic by FISH at diagnosis		0.18		0.65
Standard-risk	Ref.		Ref.	
High-risk	1.39 (0.85–2.23)		1.14 (0.62–2.12)	
Extramedullary disease		0.01		0.06
Yes	1.94 (1.15–3.26)		1.80 (0.97–3.32)	
No	Ref.		Ref.	
Previous ASCT		0.22		0.76
Yes	1.40 (0.81–2.47)		0.89 (0.44–1.82)	
No	Ref.		Ref.	
Number of prior regimens	1.33 (1.06–1.66)	0.01	1.42 (1.09–1.85)	0.009
Treatment		0.69		0.21
DPd	Ref.		Ref.	
DVd	1.11 (0.65–1.89)		0.65 (0.32–1.29)	
Double refractory		0.31		0.47
Yes	1.28 (0.78–2.12)		1.25 (0.67–2.31)	
No	Ref.		Ref.	
Response to treatment (DPd, DVd)		<0.001		0.003
sCR/CR	Ref.		Ref.	
VGPR/PR	4.47 (2.54–7.88)		3.01 (1.46–6.24)	

PFS, progression-free survival; OS, overall survival; R-ISS, revised international staging system; ASCT, autologous stem cell transplantation; DPd, Daratumumab-pomalidomide-dexamethasone; DVd, Daratumumab-bortezomib-dexamethasone; sCR/CR, stringent complete response/complete response; VGPR/PR, very good partial response/partial response; Ref., reference.

## Data Availability

The data that support the findings of this study are available on request from author, AA. The data are not publicly available due to local IRB restrictions.
